# Allergic Contact Dermatitis to Acrylates: A Case Report

**DOI:** 10.7759/cureus.66944

**Published:** 2024-08-15

**Authors:** Ovidiu Berghi, Ana Carbunaru, Liliana G Popa

**Affiliations:** 1 Allergy and Clinical Immunology, Colentina Clinical Hospital, Bucharest, ROU; 2 Dermatology, Clinica Sfanta Maria, Bucharest, ROU; 3 Dermatology, "Carol Davila" University of Medicine and Pharmacy, Bucharest, ROU

**Keywords:** allergy, pads, nails, contact dermatitis, acrylates

## Abstract

(Meth)acrylates are a common cause of allergic contact dermatitis (ACD) that can be found in medical (such as pads) and aesthetic (specifically nail cosmetics) products. Prolonged and intimate contact with sanitary pads can predispose individuals to ACD triggered by a variety of allergens. This report presents the case of a young female patient with episodes of nail contact dermatitis followed by a protracted history of intensely symptomatic allergic contact dermatitis affecting the external genital area, precipitated by methacrylates present in menstrual pads.

## Introduction

(Meth)acrylates are recognized as a common cause of allergic contact dermatitis (ACD) that can be found in various industrial, consumer, medical (such as pads), dental, cosmetic, and aesthetic (specifically nail cosmetics) products [1]. The unique characteristics of the female genital area, characterized by moisture, exposed mucous membranes, and hair follicles, coupled with prolonged and intimate contact with sanitary pads, can predispose individuals to ACD triggered by a variety of allergens, including preservatives [2], fragrances [3], and (meth)acrylates [4].

The external genital region in females frequently serves as a site for chronic eczema, presenting with persistent pruritus, a burning sensation, and often dyspareunia, significantly impacting patients' quality of life. Diagnosis can prove challenging, given the differential consideration of various infections (notably candidiasis and scabies), inflammatory conditions (such as lichen simplex chronicus, lichen planus, lichen sclerosus et atrophicus, contact dermatitis, and psoriasis), and preneoplastic and neoplastic disorders (including vulvar dysplasia, Bowen disease, and extramammary Paget disease) [5]. After excluding other potential causes, dermatologists and gynecologists should consider referring patients for allergological examination upon clinical suspicion of contact dermatitis. Avoidance of contact with the incriminated allergen is crucial for achieving complete remission [5].

This report presents the case of a young female patient with a protracted history of intensely symptomatic allergic contact dermatitis affecting the external genital area, precipitated by methacrylates present in menstrual pads. We aim to delineate optimal management strategies for similar cases.

## Case presentation

**Figure 1:**
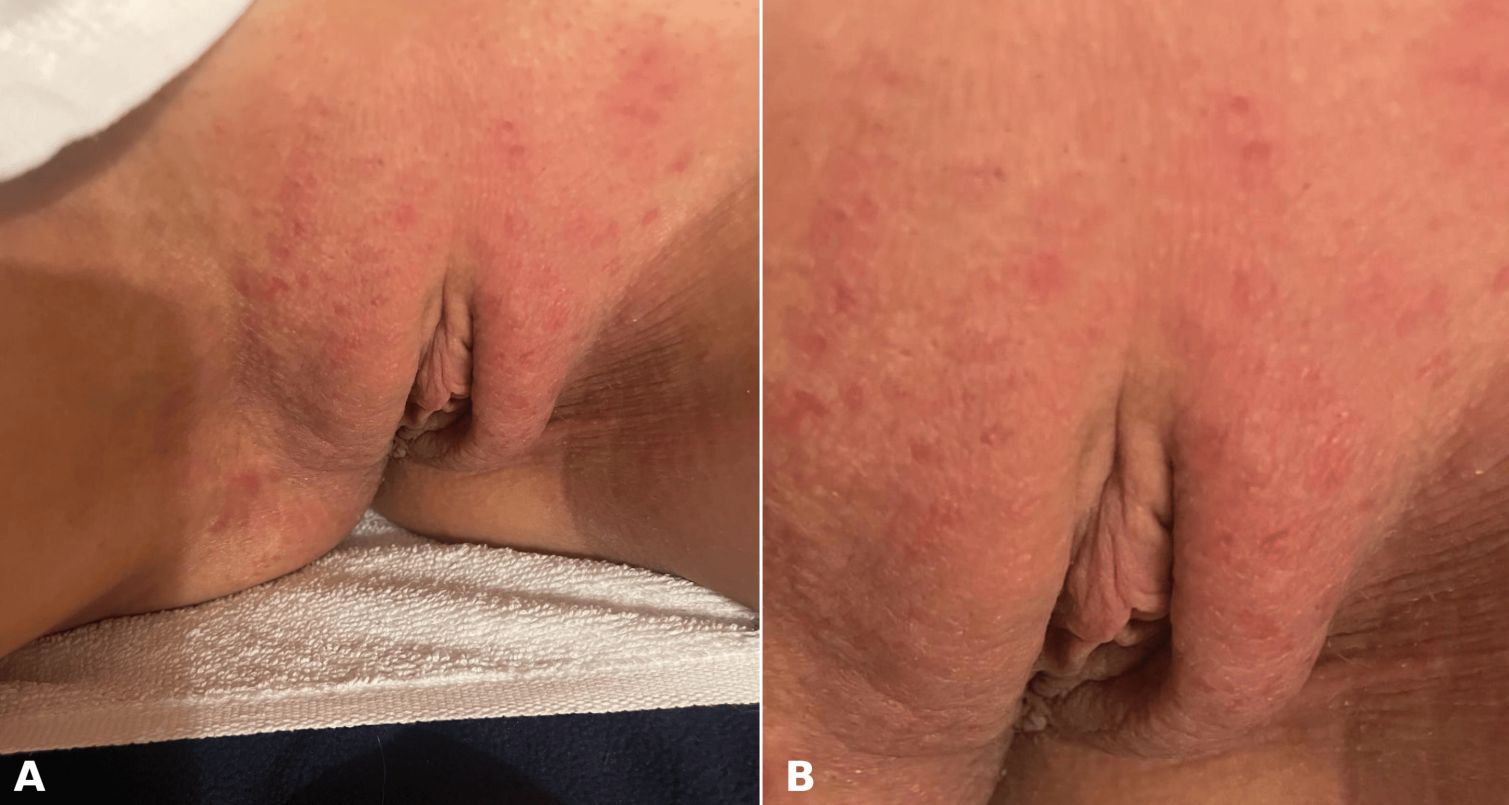
A: Chronic eczema affecting the genital area that came into contact with menstrual pads. B: Close-up image showing erythema, vesicles, and papules affecting the genital region.

A 36-year-old female, with no significant medical history, presented to our clinic with chronic dermatitis affecting the external genital area. The onset of the skin lesions occurred one year prior while using menstrual pads. Initially, the lesions were limited to the periods when pads were used. However, over subsequent months, they became chronic (Figure 1). She was evaluated by a dermatologist, who established the diagnosis of chronic dermatitis, ruling out other dermatoses.

Despite daily application of a combination topical treatment comprising betamethasone dipropionate, clotrimazole, and gentamicin for several months, the patient experienced no clinical improvement. The eczematous lesions and accompanying symptoms, including itching, pain, and burning sensation, progressively worsened, leading to considerable local discomfort throughout the day and disrupting the patient's sleep. Based on the anamnesis, the clinical picture, the lack of response to treatment, and the absence of comorbidities, concomitant medication, or other eliciting factors, the suspicion of allergic contact dermatitis was raised, and the patient was referred to our allergy clinic for further investigations.

**Figure 2:**
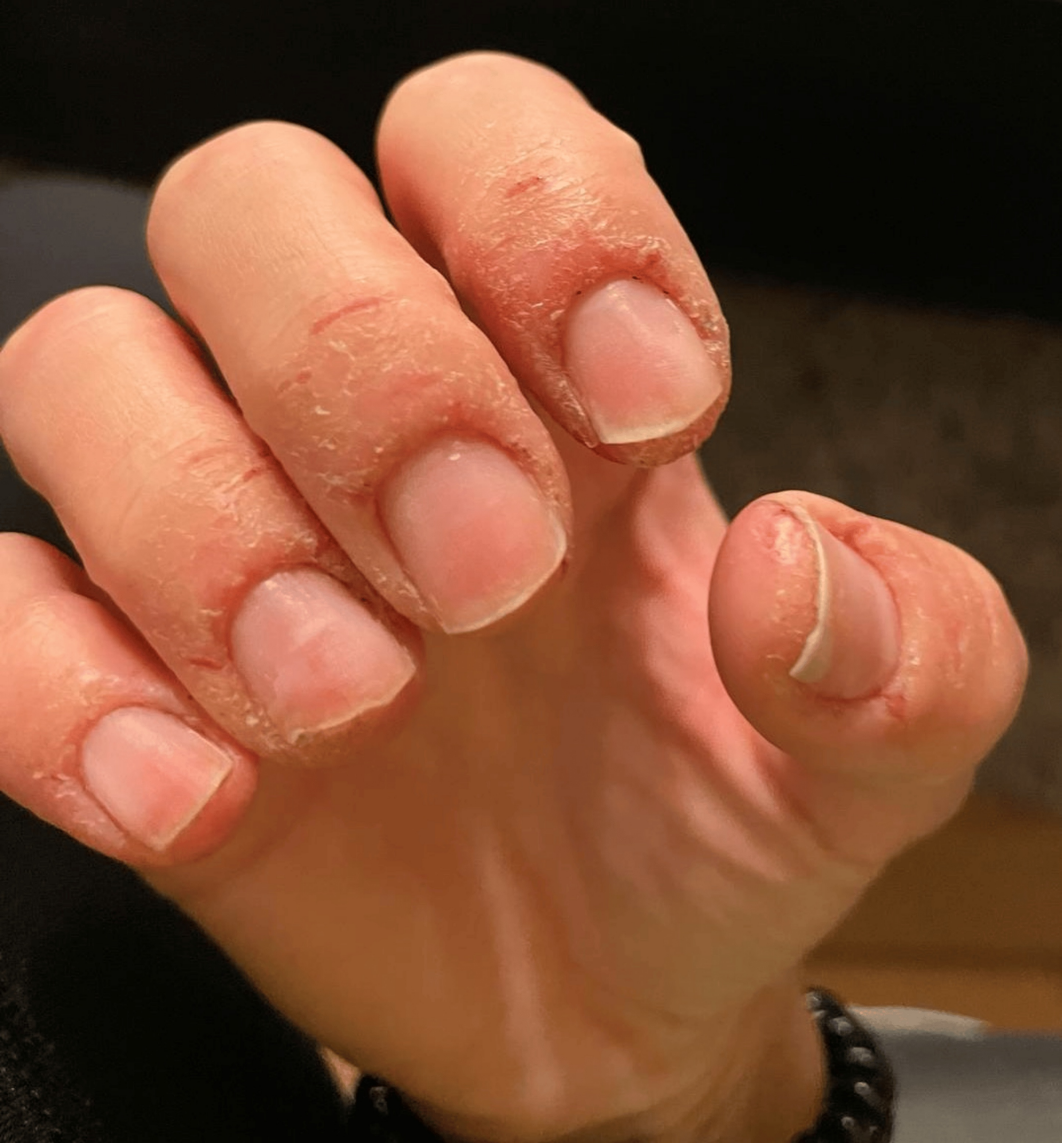
Periungual eczema after using nail polish.

The allergological anamnesis revealed a history of periungual eczema following nail polish use two years previously (Figure 2). The patient is an active adult woman, employed in an office environment devoid of exposure to toxic chemicals or hazardous substances.

**Figure 3:**
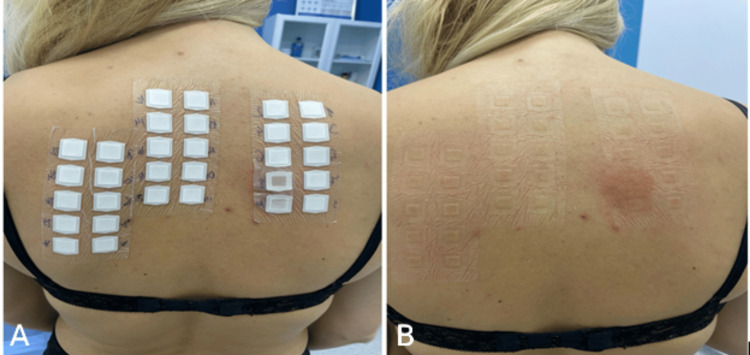
A: Patch test using the European Baseline Series S-1000 from Chemotechnique Diagnostics. B: Positive patch test results (+++) for HEMA 2% pet at 48 hours. HEMA: 2-hydroxyethyl methacrylate

**Figure 4:**
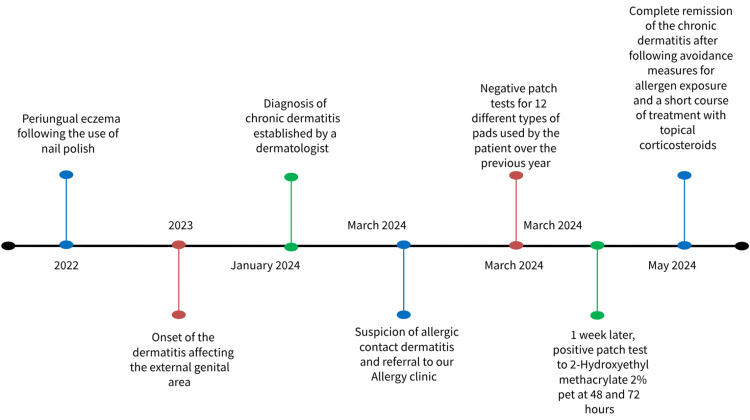
Diagram illustrating the chronology of the events.

We conducted two series of patch tests. Initially, we tested 12 different types of pads that the patient had used over the past year, including Enroush, Siempre protej slip (five variants), Bella white protej sleep, Always Cotton, Always Normal, Tadam, O.B. Always, and O.B. Organic. The pads were applied unmoistened directly on the skin. The results were negative at 48 and 72 hours. One week later, we performed a second patch test using the European Baseline Series S-1000 from Chemotechnique Diagnostics, revealing a markedly positive reaction (+++) to 2-hydroxyethyl methacrylate (HEMA) 2% pet at 48 and 72 hours (Figure 3). Throughout both sessions, the tests were occluded with IQ Ultimate Patch Test Units. Consequently, a diagnosis of allergic contact dermatitis to 2-hydroxyethyl methacrylate (HEMA) 2% pet was established. The patient received comprehensive counseling regarding avoidance measures for allergen exposure and was treated with topical corticosteroids, resulting in gradual clinical improvement. She stopped wearing any type of acrylate nail varnish. Currently, the dermatosis is in complete remission, and the patient tolerates a single type of tampon, specifically O.B. Organic. Figure 4 illustrates the chronology of the events.

## Discussion

Advancements in personal care products have largely relied on the incorporation of a wide array of chemicals. Among these, acrylates stand out as thermoplastic resins utilized across diverse products ranging from cosmetics to adhesives and industrial materials. Acrylic monomers, comprising a significant subset, exhibit potent allergenic properties, with 2-hydroxyethyl methacrylate (HEMA) emerging as a key screening allergen. While the polymerized end products are generally not deemed allergenic, they may be the cause of ACD in rare instances [6].

Pads are manufactured and marketed in various formulations. Upon conducting a comprehensive analysis of their composition, we identified numerous potential allergens present in their absorbent core and adhesive material. Notably, sodium acrylate was found in Tadam pads, unspecified acrylates were present in Bella pads, and unspecified polymers were detected in Libresse pads [7-9]. The patch test results obtained from our patient further contribute to existing evidence from prior studies, highlighting the potential for (meth)acrylate allergy associated with incontinence pads and the development of ACD following the use of acrylic nails [4].

However, our case report does have a few limitations. Firstly, we conducted direct testing of the pads on the skin, in contrast to the approach taken by Gatica-Ortega et al. [10], who opted to patch-test a moistened piece of the incontinence pads' absorbent layer. Our rationale was based on the belief that if ACD were to occur, the reaction would manifest on any part of the skin due to potential acrylate leaching.

Another limitation of our study is the utilization of the European Baseline Series, which encompasses solely HEMA. Unfortunately, due to financial constraints, we were unable to employ the specialized acrylates series tests provided by Chemotechnique Diagnostics. Nonetheless, as indicated by de Groot and Rustemeyer [1], most positive tests in patients subjected to (meth)acrylates series testing are elicited by HEMA. It is noteworthy that significant cross-allergy exists between HEMA, ethylene glycol dimethacrylate (EGDMA), and 2-hydroxypropyl methacrylate [11]. Despite these limitations, we maintain the belief that testing for HEMA in routine practice is generally adequate for diagnosing ACD to acrylates in the majority of patients.

Suuronen et al. [12] conducted an analysis of 37 nail products, revealing that 32 of them contained (meth)acrylates. Additionally, they noted significant discrepancies in the labeling of artificial nail products across the majority of items [12]. Furthermore, the prevalence of HEMA patch test positivity was found to be 2.3% in a recent multicenter European study involving 7,675 tested individuals [13]. Conversely, Marcelis et al. [2] performed a risk assessment for allergens, including α-isomethyl ionone, benzyl salicylate, hexyl cinnamaldehyde, and heliotropine, leaching from menstrual hygiene products. They concluded that while there is no risk of inducing ACD from these potential sensitizers, the authors emphasized the importance of comprehensive package labeling for all allergens, particularly for women who are already sensitized.

## Conclusions

Given the ongoing expansion of both the feminine hygiene market and the artificial nail industry, it is foreseeable that the incidence of ACD to various acrylates may rise, potentially culminating in a public health concern. Consumers should be made aware of the potential side effects associated with acrylates and their presence in everyday convenience products to mitigate the risk of further complications.
